# Dr. Charles Richards

**Published:** 1912-10

**Authors:** 


Dr. Charles Richards, Buffalo, 1904, of Sturgis, S. Dak., died of nephritis at his home, Aug. 4, aged 40.

